# Recurrent and multiple bladder tumors show conserved expression profiles

**DOI:** 10.1186/1471-2407-8-183

**Published:** 2008-06-30

**Authors:** David Lindgren, Sigurdur Gudjonsson, Kowan Ja Jee, Fredrik Liedberg, Sonja Aits, Anna Andersson, Gunilla Chebil, Åke Borg, Sakari Knuutila, Thoas Fioretos, Wiking Månsson, Mattias Höglund

**Affiliations:** 1 Department of Clinical Genetics, Lund University Hospital, Lund, Sweden; 2 Department of Urology, Lund University Hospital, Lund, Sweden; 3 Department of Pathology, Haartman Institute and HUSLAB, University of Helsinki and Helsinki University Central Hospital, Helsinki, Finland; 4 Department of Pathology and Cytology, Helsingborg Hospital, Helsingborg, Sweden; 5 Department of Oncology, Lund University Hospital, Lund, Sweden

## Abstract

**Background:**

Urothelial carcinomas originate from the epithelial cells of the inner lining of the bladder and may appear as single or as multiple synchronous tumors. Patients with urothelial carcinomas frequently show recurrences after treatment making follow-up necessary. The leading hypothesis explaining the origin of meta- and synchronous tumors assumes a monoclonal origin. However, the genetic relationship among consecutive tumors has been shown to be complex in as much as the genetic evolution does not adhere to the chronological appearance of the metachronous tumors. Consequently, genetically less evolved tumors may appear chronologically later than genetically related but more evolved tumors.

**Methods:**

Forty-nine meta- or synchronous urothelial tumors from 22 patients were analyzed using expression profiling, conventional CGH, LOH, and mutation analyses.

**Results:**

We show by CGH that partial chromosomal losses in the initial tumors may not be present in the recurring tumors, by LOH that different haplotypes may be lost and that detected regions of LOH may be smaller in recurring tumors, and that mutations present in the initial tumor may not be present in the recurring ones. In contrast we show that despite apparent genomic differences, the recurrent and multiple bladder tumors from the same patients display remarkably similar expression profiles.

**Conclusion:**

Our findings show that even though the vast majority of the analyzed meta- and synchronous tumors from the same patients are not likely to have originated directly from the preceding tumor they still show remarkably similar expressions profiles. The presented data suggests that an expression profile is established early in tumor development and that this profile is stable and maintained in recurring tumors.

## Background

Urothelial carcinomas (UC) originate from the epithelial cells of the inner lining of the bladder wall. The majority is papillary and confined to the urothelial mucosa (stage Ta) or to the lamina propria (stage T1) whereas the remaining invades the underlying muscle tissue (T2), perivesical fat (T3) or surrounding organs (T4). Most Ta tumors are of low or medium grade (G1 or G2), rarely progress, and are associated with a favorable prognosis whereas high grade Ta (TaG3) and T1 tumors represent a significant risk of tumor progression. UCs are characterized by a number of chromosomal and genetic alterations of which cytogenetic loss and loss of heterozygosity (LOH) of chromosome 9 is particularly frequent occurring in > 50% of the cases [1-3]. Furthermore, activating point mutations in the *FGFR3 *gene are found in > 70% of Ta tumors, but rarely in invasive tumors [4]. A reverse pattern is seen for *TP53*, which has led to the suggestion that UC may develop through two different genetic pathways [5]. Ta and T1 tumors are mostly treated by transurethral resection, in many cases combined with subsequent intravesical chemo- or immunotherapy. However, up to 70% of the patients have local recurrences after treatment, making follow-up by regular cytoscopy necessary. Furthermore, patients often show multiple synchronous tumors, as well as concomitant hyperplasia, dysplasia, or cancer *in situ*. Several investigations have shown that the majority, if not all, meta- and synchronous tumors are clonally related [6-8]. The leading hypotheses regarding the origin of meta- and synchronous UC include intraepithelial migration of tumor cells and intraluminal seeding from a primary carcinoma [9]. Even though these models has achieved some attention, they are at odds with the finding that genomic relationships among consecutive tumors are complex and do not adhere to a simple clonal evolution [10]. In contrast, tumors from the same patient have been shown to demonstrate similar expression profiles [11,12] indicating a similarity at the transcriptional level not seen at the genomic level. In the present investigation we address this apparent contradiction by performing extensive genomic and expressional profiling of several syn- and metachronous bladder cancers.

## Methods

### Patients and Tissues

Urothelial tumors were collected by cold-cup biopsies from the exophytic part of the bladder tumor in patients undergoing transurethral resection at the University Hospital of Lund, Sweden, between 2001 and 2005. The first resected tumor is referred to as the initial (I) tumor and the subsequent tumors as recurrences (R). The time between initial and recurring tumors ranged between 4 and 31 months. Samples were immediately transferred into transport media, transported to the laboratory and frozen at -80°C. Tumor pathology and quality (> 70% tumor cells) of tumor specimens was reviewed by one pathologist (GC). Tumor progression in stage or grade occurred in six and five patients, respectively. Regression in stage was not seen whereas a lower grade was observed in recurrent tumors from four patients. Normal urothelial tissue samples were obtained from patients in surgery for non UC-related disorders. The investigation was performed with informed consent and approved by the local ethical committee.

### Extraction of nucleic acids

Total RNA was extracted using Trizol reagent (Invitrogen, Carlsbad, CA) and purified on Qiagen RNeasy columns (Qiagen, Valencia, CA). RNA sample integrities were assessed on an Agilent 2100 Bioanalyzer (Agilent technologies, Palo Alto, CA) and samples that showed RNA degradation (RNA integrity number, RIN, less than 7) were excluded from further analyses. Genomic DNA was extracted using the DNeasy Tissue kit (Qiagen) protocol.

### Microarray hybridization and Data processing

In the present study, data from two different microarray platforms was used. Data set 1 (Ta to T1 tumors hybridized to 25 k cDNA arrays) included 15 metachronous tumors from 7 patients, tumors from 84 additional patients and 8 normal samples. Microarray hybridization and data pre- and postprocessing of tumors in Data set 1 was carried out as previously described [13]. Data set 2 (T1 to T4 tumors hybridized to 36 k oligonucleotide arrays) comprised 38 meta- or synchronous tumors from 17 patients, 91 unique tumors, and 7 normal samples. Samples in Data set 2 were hybridized to 36 k oligonucleotide arrays as described in Heidenblad et al. [14] In short, postprocessing of data in Data set 2 was performed within the Bioarray Software Environment (BASE) [15]. Spots of poor quality identified through the image analysis were removed followed by adjustment of the background corrected Cy3 and Cy5 intensities by a pin-based lowess-fit normalization algorithm. The dataset was further filtered to remove spots with median ratio values for the Cy3 or Cy5 channels lower than 15 counts. Replicate spots were averaged and features with more than 20% missing values were subsequently removed. Missing values were thereafter filled in using KNN imputation (k = 10). HCA and statistical analyses were performed using Statistica software (StatSoft, Tulsa, OK). For HCA, 1-Pearson correlation was used as distance measure and Wards' algorithm for cluster formation.

### Mutation, CGH, and LOH analyses

Mutation analyses were carried out by direct sequencing of exons 7, 10, 13, and 15 for *FGFR3 *and exons 4 to 9 for the *TP53 *gene. For samples in Data set 1, sequencing was performed on cDNA transcribed from amplified RNA as previously described [13]. For samples in Data set 2, sequencing was performed on genomic DNA using BigDye terminator chemistry (Applied Biosystems, Foster City, CA) and an ABI3700 automatic sequencer (Applied biosystems), and analyzed using the SeqScape v2.5 software (Applied Biosystems). All identified mutations were verified by reverse sequencing on an independently generated amplicon. LOH analysis for seventeen highly polymorphic microsatellite markers distributed over both arms of chromosome 9 was carried out on 36 tumors from 16 patients from which matching blood samples were available as described in Lindgren et al. [13]. Genomic profiles were obtained by conventional CGH for 35 tumors from 17 patients and performed as described by El-Rifai et al. [16]

## Results

### Mutation, CGH, and LOH analyses

We selected 49 meta- or synchronous tumors from 22 patients for molecular analyses (Table 1). Tumors from seven patients showed *FGFR3 *mutations (Table 1). In six of these, both the initial and the recurring tumors showed the same mutation. One patient (patient 43) showed different mutations in the initial and recurrent tumor. *TP53 *mutations were present in tumors from seven patients. In two patients, the recurring tumor showed a mutation that was not present in the initial tumor, and one patient (patient 31) showed a mutation in the initial tumor that was not seen in any of the three subsequent tumors. In the two synchronous tumors from patient 56, only one had a *TP53 *mutation. Hence, both *FGFR3 *and *TP53 *mutations may occur independently in meta- and synchronous tumors.

**Table 1:** Individual tumor characteristics, mutation, and CGH analyses

Tumor^1^	mtr^2^	Stage/grade^3^	FGFR3mut^4,5^	TP53mut^4^	Imbalances^6^
01.I		TaG2			dim (8p., **22p, 22q11–13.1**)
					enh (8q11–q22, Xq)

01.R1	5	TaG2			dim (8p)
					enh (8q)

02.I		T2G3			dim (19p)
					enh (1p31–p32, 1q23–q25, 8q, 12q13–q21)

02.R1	7	T2G3			enh (1q23–q24, 2p14-pter, 3p22-pter)

07.I		TaG1	S249C		na

07.R1	7	TaG2	S249C	K132N	na

09.I		T1G3		G245D	dim (**2q33-qter, 6q24-qter, **9, **10q, 11p15-pter, 17, 22**)
					enh (1p11–p21, 1q11–q31, 2q11, 2q23–q24, 3, 8q11–q22, 11q14-qter, 16p, 16q11–q13, 18p, 20, X)

09.R1	10	TIG3		G245D	dim (9)
					enh (1q22–q31, 2p13-pter, 3, 8, 16p, 16q11–q13, 20)

10.I		T1G2	S249C	R280T	dim (9q)
					enh (12q13–q21)

10.R1	5	T1G3	S249C	R280T	dim (9q22-qter, 10q24-qter)
					enh (10q21–q23, 20q, Xp, Xq11–q24)

13.I		T1G2			dim (9q, **10p12–p13**, **16p**, 17p, 19, **22**)
					enh (1p11–p31, 1q11–q41, 3, 5q14–q23, 8q13-qter, 12q15–q22, 13, 18q, 20p, X)

13.R1	6	T0			dim (4, 9, 17p, 19)
					enh (1q23-qter, 3, 5q13–q23, 13, 14, 17q, 18q, 20, X)

14.I		T1G2			dim (**8p**, 9, 10p, **17p**)
					enh (8q)

14.R1	8	T1G3			dim (9, 10p, 19)
					enh (8q)

16.I		TIG3			dim (8q)
					enh (8p, 19)

16.R1	4	T1G2			dim (8q)

18.I		T1G2			dim (8p)
					enh (1p32-pter, 1p13–p21, 1q21–q31, 1q33–q35, 3, 5p, 6p11–p22, 8q21-qter, 10q11–q23, 13q22-qter)

18.R1	16	T2G3		R273L	enh (3, 8q11–q23, 9p)

21.I		TaG2			dim (9q22-qter, 11p13-qter, 22)
					enh (12p11–p12, 12q11–q23)

21.R1	7	T1G2			dim (8p, 9q, 10p, 11p, 11q11–q13.1, 12q24-qter, 16p, 17, 18p, 19, 22)
					enh (3, 4p11–p15, 4q11–q32, 5q21–q23, 6q11–q14, 8q, 12q15–q21, 13q14-qter, 18q11–q22)

29.I		TaG2	S249C		dim (9q32-qter, **14q23-qter, 19**)
					enh (1q22-qter, 8q)

29.R1	21	TaG2	S249C		dim (9q22-qter)
					enh (1q11–q31, 13, X)

30.I		TaG2			na

30.R1	13	TaG2			na

31.I		TaG2		R290H	na

31.R1	10	TaG2			na

31.R2	17	TaG2			dim (2q34-qter, **8p**, 14, **19p**)
					enh (1q21-qter, 2p13-pter, 5p13-pter, 8q, 11q14–q24)

31.R3	19	T4G3			dim (2q31–q34, 5q11–q23, 6q, 9p12-pter)
					enh (1q23–q24, 1q32-qter, 2p, 2q11–q14, 5p, 8)

34.I		T1G3	S249C		dim (9, **11p11-pter, 12p**)
					enh (1p31–p32, 1q23–q41)

34.R1	6	T2G3	S249C		dim (8p, 9)
					enh (8q)

38.I		T1G3		R248Q	dim (9)

38.R1	9	T2G3		R248Q	na

38.R2	24	T2G3		R248Q	dim (19p)
					enh (2q14–q21, 8q22-qter)

42.I		TaG2	S249C		dim (5q, 10q11–q21, 10q23-qter, 13p, 13q11–q14, 15, 17p, 18q, 19p)
					enh (2p13-pter, 3, 5p, 6p22-pter, 8q13-qter, 9p22.1-pter, 12q14–q21.2, 18p, X)

42.R1	31	TaG1	S249C		enh (1q24-qter, 8q21–q23)

43.I		TaG1	S249C		na

43.R1	4	TaG1	Y375C		na

47.I		TaG2			na

47.R1	5	TaG2			na

47.R2	18	TaG1			na

53.I		TaG2	S249C		enh (X)

53.R1.S1	27	T1G2	S249C		dim (19p)

53.R1.S2	27	T1G2	S249C		dim (19p)
					enh (Xp11-pter, Xq26-qter)

54.S1	-	T1G3			enh (1q23)

54.S2	-	T1G3			enh (8q21-qter, 12, 13)

56.S1	-	T2G3			dim (**2q32.2-qter, 5q, 15, 16q**)
					enh (1p, 1q12–q23, 3, 5p, 8q, 13q21-qter, 16p, 17q, 20, 21, Xp22-pter)

56.S2	-	T2G3		Q331STOP	dim (8p)
					enh (1, 2p, 2q11–q21, 3, 5p, 6p12-pter, 6q21–q23, 7p, 7q32-qter, 8q, 10p, 15q21-qter, 20)

64.S1	-	T3G3	na	na	na

64.S2	-	T3G3	na	na	na

^1^I, Initial tumor, *i.e*. the first resected tumor from a given patient; R, recurrent tumor; S, synchronous tumor.

^2^mtr, months to recurrence

^3^Histopathological staging and grading were reviewed according to the 2002 TNM and 1999 WHO classification systems by one single pathologist (GC)

^4 ^Direct sequencing of exons 7, 10, and 15 of *FGFR3 *and exons 4 to 9 of *TP53 *was performed using BigDye terminator chemistry (Applied Biosystems, Foster City, CA). na, data not available either due no PCR products for sequencing or DNA quality unsuitable for CGH.

^5^FGFR3 amino acid positions are given according to the FGFR3 IIIb open reading frame; n/a, sequence data not available.

^6^Partial chromosomal losses in initial tumors incompatible with imbalances present in recurring tumors are in bold

Genomic profiles were obtained by comparative genomic hybridization (CGH) for 35 tumors from 17 patients (Table 1). Taken together, the CGH analysis indicated absence of clonal relationship in only two patients (patients 38 and 54, respectively). However, patient 38 displayed the same point mutation in *TP53 *in both samples examined, strongly suggesting a clonal relationship. Even though the vast majority of patients revealed a clonal relationship between recurring tumors, different genomic alterations were frequently observed in tumors from the same patients. In particular, nine patients (53%) showed genomic imbalance patterns in the recurrent tumors not compatible with a simple karyotypic evolution from the initial tumor (Table 1). For example, in patient 9 the initial tumor harbored partial losses of chromosome arms 2q, 6q, 10q, and 11p, which were not seen in the recurrent tumor. Likewise in patient 13, the initial tumor harbored partial losses of chromosomes 10 and 16, not seen in the recurring tumors. In addition, 8 cases (47%) showed a lower number of imbalances in the recurring than in the initial tumor.

**Figure 1:**
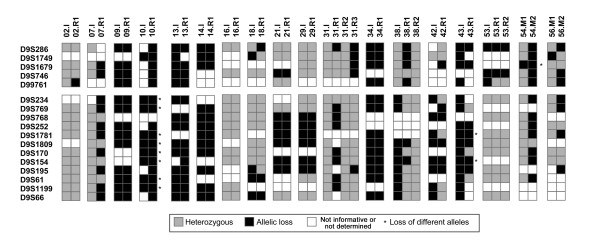
**LOH analyses of 36 tumors from 16 patients.** The markers are ordered according to their genomic position. The gap between markers indicates the location of the centromere.

LOH analyses of 17 loci on chromosome 9 were performed on 36 tumors from 16 patients (Figure 1). As for the CGH analysis, the LOH patterns also pointed to a complex genomic relationship between initial and recurring tumors not compatible with a simple genetic progression model. For example, CGH data for patient 10 indicated 9q losses in both tumors whereas the LOH analysis showed loss of different haplotypes in the initial and the recurring tumor, thereby indicating that different chromosome homologues had been lost. The synchronous tumors from patient 54 both showed LOH in the *CDKN2A*-region on 9p. However, the regions showing LOH are most likely caused by independent events as different *D9S1679 *alleles were lost in the synchronous S1 and S2 tumors, respectively. The initial and recurrent tumor from patient 43 both showed LOH on 9q. However, the region of allelic imbalance was larger in the initial than in the recurring tumor and different alleles were lost in the overlapping region at the *D9S1781 *and *D9S154 *loci. In this case, it is likely that mitotic recombination occurred in a progenitor cell prior to the genomic events leading to LOH. Altogether, the CGH and LOH analyses show that no simple karyotypic relationship seem to exist between initial and recurring or between synchronous bladder tumors.

### Gene expression profiling

**Figure 2:**
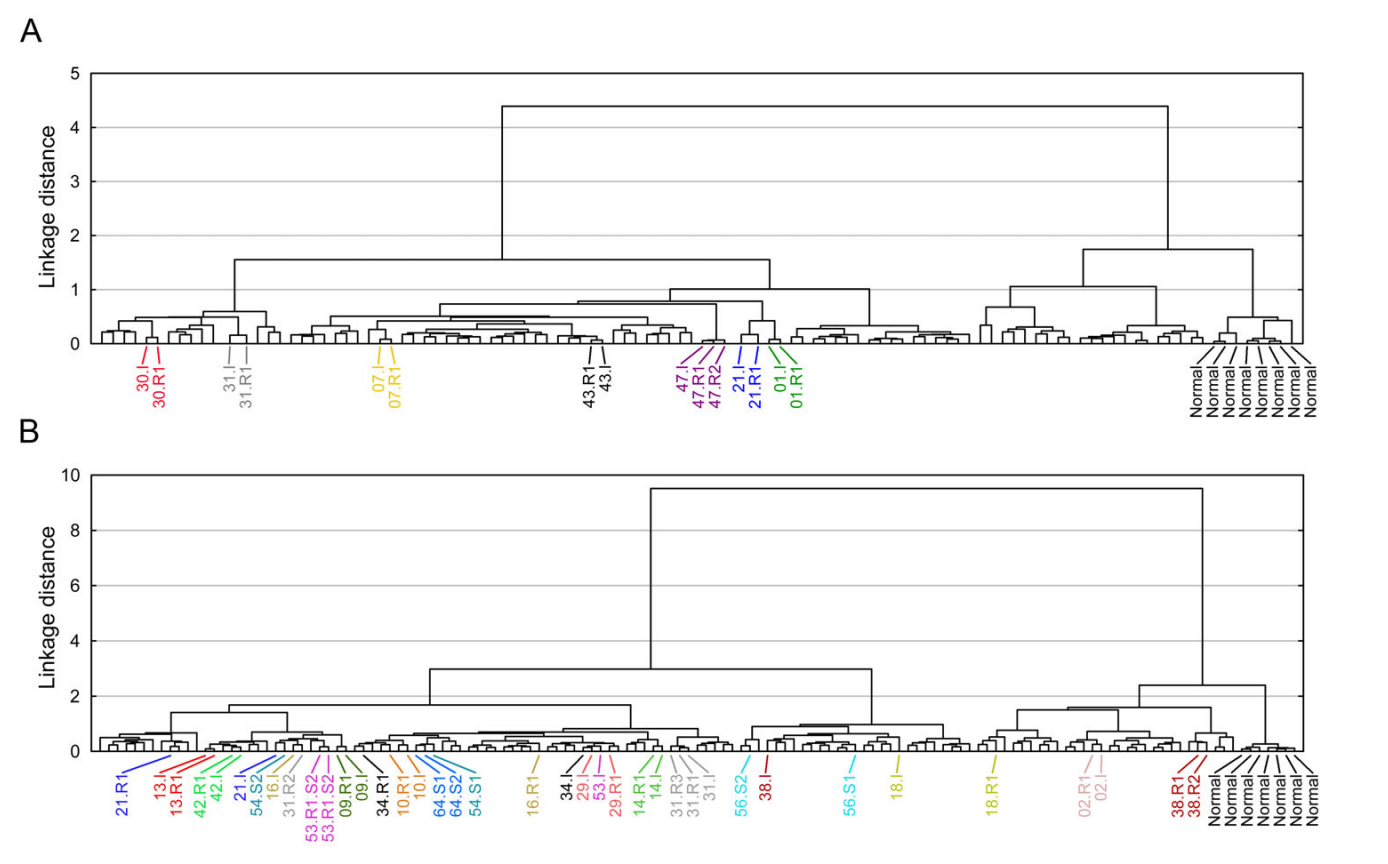
**Hierarchical cluster analysis (HCA) based on gene expression data.** A) HCA of 15 metachronous tumors from 7 patients, 84 unique tumors, and 8 normal samples hybridized to the 25k cDNA array. B) HCA of 38 meta- or synchronous tumors from 17 patients, 91 unique tumors, and 7 normal samples hybridized to the 36k oligonucleotide array. Meta- and synchronous tumors from the same patients are colored the same.

Gene expression profiling of tumors from seven of the patients was performed on a 25 k cDNA array. Expression profiles of 8 normal samples and 85 additional patients from which only one tumor sample was available (herein referred as unique tumors) were used as reference samples in a subsequently performed hierarchical cluster analysis (HCA). The HCA revealed a remarkable similarity between initial and recurrent tumors; samples from the same patient clustered adjacent to each other in all cases (Figure 2A). For the remaining 15 patients, tumor gene expression profiles were obtained using a 36 k oligonucleotide array, and these were combined with profiles from 91 unique tumors and 9 normal samples. Again, a high similarity in expression between samples from the same patients was observed (Figure 2B). Two major exceptions were however seen; initial and recurrent tumors from patients 18 and 38 clustered separately in the two main branches of the HCA dendogram. Notably, the shift in HCA branch was linked to a progression from T1 to T2 tumors in both patients. Tumors from patients 16, 34, and 54 also showed variability among the expression profiles, but to a lesser extent. In case of 34.I and 34.R1 this was associated with progression to muscle-invasive growth. Interestingly, in the tumors from patient 38 a large difference in gene expression was seen at the shift from stage T1 to T2, but the two subsequent T2 recurrences still clustered adjacent to each other in the HCA dendogram. Taken together, tumors from 18 out of 22 patients (82%) were grouped adjacent to, or in close vicinity of each other, in the HCA.

**Figure 3:**
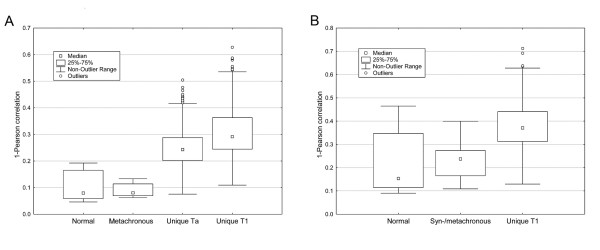
**Box plots of 1-Pearson correlation distributions.** A) 1-Pearson correlation distances among 8 normal samples, metachronous samples from 7 patients, 62 unique Ta samples, and 19 unique T1 samples estimates from expression data obtained from the 25k cDNA platform. B) 1-Pearson correlation distances among 7 normal samples, meta- or synchronous tumors from 12 patients, and 36 unique T1 tumors estimated from expression data obtained from 36k oligonucleotide platform. The calculation of distances among meta- and synchronous is based on distances among tumors originating from the same patients.

To further investigate the similarity in gene expression between tumors from the same patient, the 1-Pearson correlation was calculated between the gene expression for meta- or synchronous tumors from the same patient and compared to the 1-Pearson correlations within the group of normal and unique tumor samples, respectively (Figure 3A, and 3B). As most recurring tumors were either of stage Ta or T1, only unique Ta and T1 tumors were considered in this comparison. For the expression data obtained from the cDNA-array, the median distances were 0.25 and 0.29 within the groups of unique Ta and T1 tumors, respectively (Figure 3A). In contrast, metachronous tumors from the same patient showed a much higher correlation between samples, with a median distance of 0.09. The mean distance among metachronous tumors from the same patients was found to differ significantly from the mean distances among unique Ta or T1 tumors (p < 0.001 and p < 0.001, respectively, Mann-Whitney U-test), but not from the distances between normal samples (p = 0.6). Similar results were obtained when considering expression data obtained from the 36 k oligonucleotide platform (Figure 3B). We did not consider metachronous tumors showing progression from T1 to > T1 in this analysis. Again, the mean distances among tumors from the same patient were significantly lower as compared to the mean distance among unique tumors (p < 0.001), but not from the mean distance among normal samples (p = 0.3). For both the cDNA- and the oligonucleotide platforms, the magnitude and variation of the distances among recurrent and multiple tumors were the same as for distances among normal samples (Figure 3A and 3B), and thus, the variability observed between meta- and synchronous tumors from the same patient may largely be explained by experimental variation, if assuming normal samples from different individuals to be almost identical. Furthermore, distances among synchronous tumors from the same patients did not differ from distances among metachronous tumors (p = 0.76). The observed similarity is not likely to be caused by insufficient resection as the majority of the recurrences show genomic alterations incompatible with a simple re-growth from an initial tumor. Hence, we argue that the highly similar gene expression profiles observed for meta- and synchronous tumors is a true biological feature of recurrent and multiple tumors.

## Discussion

The observed similarity at the expression level among syn- and metachronous tumors revealed in the present investigation, and noted by others [11,12], is in contrast to the inconsistency seen at the genomic level. Even though most recurring meta- and synchronous tumors showed a clonal relationship, no simple genomic evolution from chronologically early to late tumors could be invoked. Partial chromosomal losses detected by CGH are highly informative in this respect, as these events are not easily compensated for by additional changes. Whole chromosome losses, on the other hand, may be counteracted by a subsequent duplication of the remaining homolog and gains may be lost as a part of a genomic evolution. The initial tumors in 9 of the patients showed partial chromosome losses not seen in the recurring tumors. The LOH analyses corroborated the complex genomic relationships among related recurrences but also revealed further incompatible genomic alterations not seen by CGH. *FGFR3 *mutations are particularly frequent in low grade and low stage tumors [4] and are believed to act as an early genetic change in UC development. In spite of this, one patient showed different *FGFR3 *mutations in the initial and the recurring tumor. The *TP53 *mutation pattern was also incompatible with a simple genetic progression model in 2 out of 7 patients. Clearly, meta- and synchronous tumors show differences at the genomic level incompatible with a simple genetic progression from initial to recurrent tumors.

The present findings further extend previously published results showing that the chronology of tumor presentation is not reflected in the genetic progression of the tumors [10]. These authors compared the chronology of the genetic events with the chronology of tumor appearance in patients with several recurrences and concluded that the genetic progression trees, as determined by LOH and mutation analyses, better reflected the tumor evolution than their chronologic order of presentation. Hence, as also shown in the present investigation, tumors with more evolved genomes may appear before clonally related recurrences with less evolved genomes. This suggests that pre-neoplastic cells with clonally related but differently evolved genomes may co-exist in the urothelium, and that cells in these fields may independently produce overt tumors [17,18]. This suggestion is in agreement with several histologic-genetic mapping investigations that have shown that low grade intraurothelial lesions in patients with UC share genomic and genetic changes with the adjacent tumors [19-23]. Notably, genomic imbalances and gene mutations seen in synchronous tumors may also be present in microscopically normal mucosa surrounding the tumor foci, suggesting that synchronous tumors originate from fields of genetically altered urothelium. The present findings further imply that the pre-neoplastic cells maintained within the urothelium have acquired an altered expression profile that is remarkably stable during the subsequent genomic evolution leading to meta- and synchronous Ta or T1 tumors. The formation of a fixed expression profile is thus a likely primary event in the development of UC.

## Conclusion

Our findings show that meta- and synchronous tumors in the bladder may harbor genomic alterations not compatible with a simple tumor progression model. Even though most alterations were clonal, recurring tumors are not likely to have originated directly from the preceding tumor. Compared with expression profiles for tumors in a large reference set obtained from different patients, initial and recurring tumors from the same patients showed remarkably similar expressions profiles; a similarity of the same magnitude as among normal samples taken from different individuals. The presented data suggests that an expression profile is established early in tumor development and that this profile is stable and maintained in recurring tumors.

## Competing interests

The authors declare that they have no competing interests.

## Authors' contributions

DL performed the mutation analyses; DL and SA performed the LOH analyses, DL, AA, TF and ÅB performed and were responsible for the expression profiling, KJJ and SK performed the CGH analyses, SG, FL, and WM collected the samples and were responsible for the clinical data, GC performed the pathological evaluation, DL and MH drafted the manuscript, MH conceived the investigation. All authors read and approved the final manuscript.

## Pre-publication history

The pre-publication history for this paper can be accessed here:


